# Burn wound healing and treatment: review and advancements

**DOI:** 10.1186/s13054-015-0961-2

**Published:** 2015-06-12

**Authors:** Matthew P. Rowan, Leopoldo C. Cancio, Eric A. Elster, David M. Burmeister, Lloyd F. Rose, Shanmugasundaram Natesan, Rodney K. Chan, Robert J. Christy, Kevin K. Chung

**Affiliations:** United States Army Institute for Surgical Research, 3698 Chambers Pass, Fort Sam Houston, TX 78234 USA; Uniformed Services University of the Health Sciences, 4301 Jones Bridge Rd, Bethesda, MD 20814 USA; Brooke Army Medical Center, 3551 Roger Brook Dr, Fort Sam Houston, TX 78234 USA

## Abstract

Burns are a prevalent and burdensome critical care problem. The priorities of specialized facilities focus on stabilizing the patient, preventing infection, and optimizing functional recovery. Research on burns has generated sustained interest over the past few decades, and several important advancements have resulted in more effective patient stabilization and decreased mortality, especially among young patients and those with burns of intermediate extent. However, for the intensivist, challenges often exist that complicate patient support and stabilization. Furthermore, burn wounds are complex and can present unique difficulties that require late intervention or life-long rehabilitation. In addition to improvements in patient stabilization and care, research in burn wound care has yielded advancements that will continue to improve functional recovery. This article reviews recent advancements in the care of burn patients with a focus on the pathophysiology and treatment of burn wounds.

## Introduction

Acute thermal injuries requiring medical treatment affect nearly half a million Americans each year, with approximately 40,000 hospitalizations and 3,400 deaths annually [1]. The survival rate for admitted burn patients has improved consistently over the past four decades [2] and is currently a favorable 97 % for patients admitted to burn centers [3]. This can be largely attributed to national decreases in burn size, improvements in burn critical care, and advancements in burn wound care and treatment that have been driven by research, as reflected in the dramatic increase in burn publications over the last several decades [4, 5]. Since the first International Congress on Research in Burns over 50 years ago, progress has been made in a host of areas, and vital improvements in early resuscitation, infection management, wound excision and coverage, and fluid management have helped in the fight against burn mortality [6, 7]. This review presents an update on the care of burn patients, with special emphasis on the mechanisms underlying burn wound healing and recent advancements in burn wound care.

## Pathophysiology of burn wounds

Thermal burns from dry sources (fire or flame) and wet sources (scalds) account for approximately 80 % of all reported burns [8] and can be classified based on the depth of burn [9, 10]. In addition to local injury at the site of burn, severe thermal injury over a large area of the skin, roughly 20 % total body surface area (TBSA) or greater, results in acute systemic responses collectively known as burn shock [11]. Burn shock is characterized by increased capillary permeability, increased hydrostatic pressure across the microvasculature, protein and fluid movement from the intravascular space into the interstitial space, increased systemic vascular resistance, reduced cardiac output, and hypovolemia requiring fluid resuscitation [12]. The edema that forms in the interstitial space forms rapidly in the first 8 h following burn injury, and continues to form more slowly for at least 18 h [13]. Volume requirements for resuscitation can be estimated by the total burn size and the patient’s weight (or body surface area). Additional factors influencing these needs include the presence or absence of inhalation injury, the extent of full-thickness burns, and the time since injury [12]. The actual fluid infusion rate is then titrated hourly, based on the adequacy of physiological responses, such as the urine output [14].

Following successful resuscitation, patients with larger burns then enter a more prolonged period of hypermetabolism, chronic inflammation, and lean body mass wasting, all of which may impair wound healing [15]. Additionally, an increased susceptibility to infection due to altered immune status may lead to sepsis, further exacerbating systemic inflammation [16]. Sustained hypermetabolism and inflammation impair wound healing through delayed re-epithelialization [17, 18]. The extent of inflammation and hypermetabolism is related to the extent [19] and depth of burn, as deeper burns show higher levels of circulating cytokines [20] and a greater hypermetabolic response [21]. Similarly, the extent of burn is an efficient predictor of hospital length of stay [19, 22] and mortality [19, 23].

According to one model, the burn wound can be divided into three zones based on the severity of tissue destruction and alterations in blood flow [10, 24–26]. The central part of the wound, known as the zone of coagulation, is exposed to the greatest amount of heat and suffers the most damage. Proteins denature above 41 °C (106 °F), so excessive heat at the site of injury results in extensive protein denaturation, degradation, and coagulation, leading to tissue necrosis. Around the central zone of coagulation is the zone of stasis, or zone of ischemia, which is characterized by decreased perfusion and potentially salvageable tissue [10]. In this zone, hypoxia and ischemia can lead to tissue necrosis within 48 h of injury in the absence of intervention [27]. The mechanisms underlying apoptosis and necrosis in the ischemic zone remain poorly understood, but appear to involve immediate autophagy within the first 24 h following injury and delayed-onset apoptosis around 24 to 48 h postburn [27]. Other studies have shown apoptosis to be active as early as 30 min postburn [28] depending on the intensity of the burn injury [29]. Oxidative stress may play a role in the development of necrosis, as preclinical studies have demonstrated promising reductions in necrosis with systemic antioxidant administration [30]. At the outermost regions of the burn wound is the zone of hyperemia that receives increased blood flow via inflammatory vasodilation and will likely recover, barring infection or other injury [25].

Although burns are different from other wounds in some respects, such as the degree of systemic inflammation [31], healing of all wounds is a dynamic process with overlapping phases [32] (Table 1). The initial inflammatory phase brings neutrophils and monocytes to the site of injury via localized vasodilation and fluid extravasation, thereby initiating an immune response that is later sustained by the recruitment of macrophages by chemokines [31]. The inflammatory phase serves not only to prevent infection during healing, but also to degrade necrotic tissue and activate signals required for wound repair [33]. Following, and overlapping with the inflammatory response, the proliferative phase is characterized by keratinocyte and fibroblast activation by cytokines and growth factors [34]. In this phase, keratinocytes migrate over the wound to assist in closure and restoration of a vascular network, which is a vital step in the wound healing process [35]. This network of communication between stromal, endothelial, and immune cells determines the course of healing, including closure and revascularization.

**Table 1:** Phases of wound healing

Phase	Characteristics	Key players
Inflammatory	Vasodilation	Neutrophils
	Fluid extravasation	Monocytes
	Edema	Macrophages
Proliferative	Wound closure	Keratinocytes
	Revascularization	Fibroblasts
Remodeling	Wound maturation	Collagen
	Scarring	Elastin
		Fibroblasts/myofibroblasts

Overlapping with the proliferative phase, the final phase of healing involves remodeling the wound [36]. During the remodeling phase, the wound scar matures [31] as collagen and elastin are deposited and continuously reformed as fibroblasts become myofibroblasts [37]. Myofibroblasts adopt a contractile phenotype, and thus are involved in wound contracture [38]. The conversion from fibroblasts to myofibroblasts controls a delicate balance between contraction and re-epithelialization that, in part, determines the pliability of the repaired wound [39]. In addition to fibroblast conversion, apoptosis of keratinocytes and inflammatory cells are key steps in the termination of wound healing and the overall final appearance of the wound [40].

## Optimization of burn wound healing

### Inflammation

Inflammation is vital to successful burn wound healing, and inflammatory mediators (cytokines, kinins, lipids, and so forth) provide immune signals to recruit leukocytes and macrophages that initiate the proliferative phase [37]. Wound re-epithelialization, or closure, in the proliferative phase via keratinocyte and fibroblast activation, or migration from dedifferentiated hair follicles and other epidermal analogs [41, 42], is mediated by cytokines recruited in the inflammatory phase. While this indicates that inflammation is essential for wound healing, aberrant inflammatory pathways have also been linked to hypertrophic scarring, and anti-inflammatory treatments could potentially aggravate symptoms and delay wound healing [40, 43, 44].

Significant edema that is initiated by several factors including vasodilation, extravascular osmotic activity, and increased microvascular permeability often accompanies inflammation [45]. Excessive or prolonged edema and inflammation exacerbate pain and impair wound healing [17, 18]. Interestingly, studies suggest that in the absence of infection, inflammation might not be required for tissue repair [46]. Since inflammation can have both beneficial and detrimental effects on burn wound healing, the clinical challenge becomes management, applying therapeutic intervention only when inflammation and edema become excessive.

Treatment of inflammation in large burns is difficult, as recently discussed in detail elsewhere [16]. Traditional anti-inflammatory treatments that focus on the inhibition of prostaglandin synthesis, such as nonsteroidal anti-inflammatory drugs or glucocorticoids, impair wound healing [47]. However, steroid administration has been shown to reduce inflammation, pain, and length of hospital stay in burn patients in several small studies [48, 49]. Early excision and grafting has become the gold standard for treatment of full and deep partial thickness burns [50, 51], in part because early excision helps reduce the risk of infection and scarring [52–54]. The timing of debridement coincides with the inflammatory phase of healing, as the burn eschar removed during excision is an inflammatory nidus and a rich pabulum for bacterial proliferation.

Nontraditional anti-inflammatory treatments, such as opioids, have gained considerable attention but have yet to translate promising preclinical results into clinical practice for wound healing. While the majority of animal studies have demonstrated consistent anti-inflammatory effects of opioids on peripheral neurons [55], clinical studies have shown little to no effect on inflammation [56]. Furthermore, topical morphine delayed the early inflammatory phase and accelerated the later proliferative phase [57, 58], which is supported by in vitro studies showing opioid stimulation of keratinocyte migration [59]. Large-scale clinical trials evaluating opioid efficacy on wound healing have not yet been conducted [60].

### Infection

The skin functions as a barrier to the external environment to maintain fluid homeostasis and body temperature, while providing sensory information along with metabolic and immunological support. Damage to this barrier following a burn disrupts the innate immune system and increases susceptibility to bacterial infection [61]. Burn wound infection was defined in a rat model with *Pseudomonas aeruginosa* [62, 63], in which the following progression was observed: burn wound colonization; invasion into subjacent tissue within 5 days; destruction of granulation tissue; visceral hematogenous lesions; and leukopenia, hypothermia, and death. Burn patients are at high risk for infection [64], especially drug-resistant infection [65], which often results in significantly longer hospital stays, delayed wound healing, higher costs, and higher mortality [66]. Infection can lead to the development of a pronounced immune response, accompanied by sepsis or septic shock, which results in hypotension and impaired perfusion of end organs, including the skin – all processes that delay wound healing. Furthermore, the leading causes of death following a severe burn are sepsis and multiorgan failure [67–69], so prevention and management of infection is a primary concern in the treatment of burn patients. Early and accurate diagnosis of infection is difficult: C-reactive protein and the white blood cell count are most often used, since the diagnostic power of procalcitonin is questionable in burns [70]. Consensus definitions of sepsis and infection have recently been proposed that are more relevant to the burn population and are often used clinically but still require validation [71].

The management of burn wound infections has been extensively reviewed elsewhere [61, 64–66, 72–77]. Since the adoption of topical antibiotics, such as mafenide in the 1960s and silver sulfadiazine in the 1970s, and of early excision and grafting in the 1970s and thereafter, systemic infections and mortality have consistently decreased [68, 72, 78]. However, Gram-positive and Gram-negative bacterial infections still remain one of the most common causes of mortality following burn injury [73]. Bacterial cultures can aid in the selection of an appropriate antibiotic, especially in cases of bacterial drug resistance, but altered pharmacokinetic parameters in burn patients must be considered and dosing should be adjusted accordingly to maximize antibiotic efficacy [79]. Importantly, effective topical antimicrobials do not exist for invasive fungal infections, and fungal wound infections are associated with greater mortality rates in large burns (>30 % TBSA) [80]. Owing to high lethality, suspicion of an invasive burn wound infection mandates rapid diagnosis, often by histopathology, and excision or re-excision of the wound.

### Nutrition

Sustained hypermetabolism, hormone elevations, and muscle wasting following severe burn injury all contribute to the clinical outcome, with magnitude and duration that are unique to burns [81, 82]. Accordingly, reducing the impact of a hypermetabolic state and providing adequate nutrition are key factors that affect burn wound healing and recovery [83], as has been reviewed elsewhere [84]. There is a difficult balance between the additional caloric needs to meet the demand from hypermetabolism and the consequences of nutrient overconsumption. Nutritional support following a burn injury is a complex issue. For example, early excision and aggressive feeding in children does not diminish energy expenditure but is associated with decreased muscle protein catabolism, a decreased rate of burn sepsis, and significantly lower bacterial counts from excised tissue [85]. In adults, early nutritional support is correlated with shorter stays, accelerated wound healing, and decreased risk of infection [86].

Several nutritional factors must be considered. For example, excess carbohydrate consumption may lead to hyperglycemia [87] that can exacerbate systemic inflammation and muscle degradation [88, 89]. Furthermore, excess fat consumption may exaggerate the immunosuppressed state [90]; and since major burn injuries may also result in immunosuppression [91], this exaggeration may increase the risk for infection and sepsis. Carbohydrate and fat intake must therefore be closely monitored in burn patients. Guidelines for nutritional support of burn patients vary, but consensus recommendations have been given by the American Burn Association and the American Society for Parenteral and Enteral Nutrition for carbohydrates, proteins, and fats [84].

In addition to support with amino acids and vitamins [84], administration of insulin has been shown to decrease healing time by reducing protein catabolism and increasing skeletal muscle protein synthesis [92–96]. More research is needed to optimize insulin delivery, as many recombinant growth factors, such as epidermal growth factor and transforming growth factor, are often cost prohibitive [93]. Other anabolic agents, such as oxandrolone, have been shown to increase lean body mass recovery, decrease length of stay, and improve overall outcomes, including wound healing [97–100]. Additionally, while conventional theory suggests that hemoglobin levels must be maintained above 10 g/dl to promote wound healing [101], preliminary evidence suggests that mild to moderate anemia has no effect on graft success if perfusion is maintained with proper circulatory volume [102]. The results of a multicenter, randomized, controlled trial (ClinicalTrials.gov NCT01079247) comparing blood transfusion with lower volumes (target hemoglobin of 7 to 8 g/dl) and conventional volumes (target hemoglobin >10 g/dl) for a large cohort of patients are expected soon and will allow for more definitive clinical guidelines on blood transfusion volumes.

### Resuscitation

Severe thermal injuries over a large area of the skin (>20 % TBSA) require fluid resuscitation for stabilization. Although volume guidelines and fluid compositions vary widely between centers, the goal of fluid resuscitation is to maintain organ perfusion with the least amount of fluid necessary [12]. Common traditional resuscitation formulas, such as the modified Brooke, and Parkland formulas, employ crystalloids such as lactated Ringer’s that contain sodium, chloride, calcium, potassium, and lactate. During large-volume resuscitations, the addition of colloids (for example, albumin, fresh frozen plasma) as adjuncts has been successful in reducing the total volume [12]. Despite extensive research into resuscitation fluid compositions and volumes, little is known about the effect of resuscitation on wound healing. A recent meta-analysis showed a positive association between the number of grafting procedures and hypernatremia, suggesting that high serum sodium levels may inhibit graft take [103]. Additionally, we have recently shown that the rate of wound closure (healing rate) is significantly faster in patients who received lower 24-h fluid resuscitation volumes [104]. More work is needed to evaluate the effect of resuscitation on wound healing trajectories before clinical recommendations for preferred fluid compositions and volumes can be made.

### Wound coverage and grafting

Early excision and grafting has been the standard of care for decades. Most studies have shown that excision within 24 to 48 h after injury is associated with decreased blood loss, infection, length of hospital stay and mortality, and increased graft take [105–108], although mortality reductions may only occur in patients without inhalation injury [109]. Since one of the main challenges in treating acute thermal injuries is preventing infection, excising the eschar and covering the wound as early as possible are critical. The standard for rapid and permanent closure of full-thickness burns is a split-thickness skin graft from an uninjured donor site on the same patient (autograft). Such grafting provides sufficient coverage without risk of rejection, although meta-analyses have yet to determine the failure rate of split-thickness skin grafts in burn patients. Split-thickness skin grafts can be meshed with variable expansion ratios to increase the coverage area, but concerns remain over the effect that meshing has on range of motion [110] and the graft site healing rate. On the other hand, donor sites are painful and impose their own wound-healing burden on the patient [111]. Various dressings have been used to cover donor sites during healing, with variable results [112].

Patients with more extensive burns often require temporary coverage with an allograft, xenograft, skin substitute, or dermal analog due to insufficient or unavailable donor sites. Allografts, or tissue taken from a living or deceased human donor, and xenografts, taken from a different species, promote re-epithelialization and prepare the wound bed for autograft, increasing the healing rate when compared with traditional dressings [113]. A recent meta-analysis suggested that since allografts and xenografts appear to be equally effective, xenografts may be a superior choice for their increased safety and reduced price [114]. However, caution should be exercised in drawing broad conclusions from this meta-analysis because the cited studies lack standardization and critical details such as depth and size of burn, and many studies cited were merely anecdotal. A cadaver allograft is thus widely considered the best material for temporary closure of excised wounds in patients with extensive, life-threatening burns and inadequate donor sites. The cadaver allograft is also the preferred material for protection of widely meshed autografts (3:1 or higher meshing ratios) during healing. In the latter setting, the allograft is applied over the meshed autograft in the manner of a sandwich.

A variety of different skin substitutes and dermal analogs exist [115–119] (Table 2) that can be broadly divided into those which replace the epidermis or replace the dermis [120, 121]. Epidermal substitutes are normally only a few cell layers thick and lack normal dermal components [122, 123]. Commercially available dermal substitutes include acellular matrices, commonly from human – for example, Alloderm (LifeCell, Bridgewater, NJ, USA) or GraftJacket (KCI, San Antonio, TX, USA) – or other sources (for example, Integra; Integra LifeSciences, Plainsboro, NJ, USA). Biobrane (Smith & Nephew, London, UK) is a semisynthetic, bilaminar material consisting of a nylon-mesh dermal analog (bonded with porcine collagen) and a silicone epidermal analog. Biobrane is used for temporary closure of superficial burns and donor sites [124, 125]. Products currently under development integrate the concept of dermal scaffolds that actively promote revascularization by incorporating stem cells and growth factors to recreate a favorable cellular microenvironment [126, 127].

**Table 2:** Skin substitutes and coverage options

Product name	Classification	Characteristics	Availability (company)
EpiDex	Autologous	Keratinocyte-based	No (Modex, Lausanne, Switzerland)
Alloderm	Acellular	Human origin	Yes (LifeCell, Bridgewater, NJ, USA)
		Dermal matrix	
GraftJacket	Acellular	Human origin	Yes (KCI, San Antonio, TX, USA)
		Tissue scaffold	
Integra	Acellular	Bovine/shark origin	Yes (Integra, Plainsboro, NJ, USA)
		Bilayer matrix	
Biobrane	Acellular	Biocomposite dressing, nylon fibers in silicone with collagen	Yes (Smith & Nephew, London, UK)
Dermagraft	Cellular	Bioabsorbable polyglactin mesh scaffold with human fibroblasts (neonatal origin)	Yes (Organogenesis, Canton, MA, USA)
Epicel	Cellular	Keratinocyte-based cultured epidermal autograft	Yes (Genzyme, Cambridge, MA, USA)
Recell	Cellular	Autologous cell suspension of keratinocytes, fibroblasts, Langerhans cells and melanocytes	Yes (Avita, Northridge, CA, USA)
		Sprayable after culture	

Numerous options exist for dressings [128, 129]. The selection of an appropriate dressing depends on several factors, including depth of burn, condition of the wound bed, wound location, desired moisture retention and drainage, required frequency of dressing changes, and cost. While many factors must be considered in dressing selection, the goals in selecting the most appropriate dressing should include providing protection from contamination (bacterial or otherwise) and from physical damage, allowing gas exchange and moisture retention, and providing comfort to enhance functional recovery. The traditional approach to burn wound care developed at the US Army Burn Center includes alternation of mafenide acetate cream in the morning and silver sulfadiazine cream in the evening, with gauze dressings used over the creams. More recently, silver-impregnated and other dressings have been introduced. Major classes of dressings include: alginate, for example Aquacel (ConvaTec, Bridgewater, NJ, USA), Comfeel (Coloplast, Minneapolis, MN, USA), or Sorbsan (Mylan, Morgantown, WV, USA); antimicrobial, for example Acticoat (Smith & Nephew, London, UK) or Silverlon (Argentum, Geneva, IL, USA); collagen, for example Fibracol (Johnson & Johnson, New Brunswick, NJ) or Puracol (Medline, Mundelein, IL, USA); hydrocolloid, for example Duoderm (ConvaTec, Bridgewater, NJ, USA), Granuflex (ConvaTec, Bridgewater, NJ, USA), or Tegaderm (3M, Maplewood, MN, USA); hydrogel, for example Dermagel (Maximilian Zenho & Co, Brussels, Belgium), SilvaSorb (Medline, Mundelein, IL, USA), or Skintegrity (Medline, Mundelein, IL, USA); and polyurethane foam, for example Allevyn (Smith & Nephew, London, UK) or Lyofoa (Molnycke, Gothenburg, Sweden). Notably, many of these dressings exhibit antimicrobial properties through silver impregnation, but recent studies suggest silver may delay wound healing and should not be routinely used on uninfected donor skin [130, 131] even though silver dressings may reduce wound pain [132]. In patients with extensive or deep burns, antimicrobial efficacy should be the first priority in burn wound care.

Alternatively, cell-based techniques for more permanent coverage have made progress. Research on cultured epithelial cells has made advancements, especially with respect to culture time. Culture-based options, such as Epicel (Genzyme, Cambridge, MA, USA), use a small biopsy of the patient’s skin to provide keratinocytes, which are expanded over 2 to 3 weeks (for Epicel, in the presence of proliferation-arrested murine fibroblasts) into a confluent epidermal autograft. Other options, such as ReCell (Avita, Northridge, CA, USA), take a small biopsy of the patient’s skin and prepare a mixture of keratinocytes, melanocytes, and stem cells in a liquid formulation for spraying onto the excised burn wound during the same operation [133–135]. These techniques may reduce the amount of donor skin needed for treatment of large burns, significantly reducing the healing time of both the donor and the burn sites, and increasing overall graft success and scar quality [136]. More work is needed on cell-based coverage options before widespread implementation can be recommended.

### Keratinocytes and stem cells

As mentioned previously, keratinocytes play a vital role in wound closure. Cytokine activation causes keratinocyte migration in the proliferative phase, leading to closure and restoration of a vascular network [35]. Keratinocytes can also be activated by mu opioid receptor agonists [59] but the role of these agonists on inflammation and wound closure remains unclear [57, 58]. Despite positive studies with EpiDex (Modex, Lausanne, Switzerland) – an engineered, fully differentiated autologous skin substitute derived from keratinocytes showing efficacy comparable with split-thickness skin grafts in wound closure and healing [137] – results have yet to translate into clinically viable options. Studies evaluating expansion of keratinocytes on human fibroblasts following trypsin extraction [138], and using engineered skin with keratinocytes on a fibrin matrix [139], have demonstrated improvements in wound healing. Retrospective analyses on autologous keratinocytes showed that cultured allogeneic or autologous keratinocytes may accelerate wound healing [140, 141]. Taken together, the future impact of keratinocyte-mediated cell coverage options is promising, but more research is needed [134]. Additionally, keratinocyte-based treatments should be pursued carefully, as overactivation of keratinocytes can contribute to the development of hypertrophic scarring [43, 142].

The use of adult stem cells, including bone marrow stem cells, hair follicle stem cells, and adipose stem cells, in acute burn care is an exciting topic [143]. Addition of bone marrow stem cells to nonhealing chronic wounds leads to engraftment of cells and enhanced wound healing [144, 145]. Moreover, studies have reported that bone marrow stem cells can transdifferentiate towards multiple skin cell types [146]. Mechanisms of action of bone marrow stem cells in burns are not fully elucidated, but modulation of inflammation has occurred after radiation burns in humans [147]. Similarly, adipose stem cells accelerate re-epithelialization by paracrine activation of host cells via growth factor secretion [148, 149]. Also, hair follicle stem cells are capable of generating a stratified epidermis on human burn wounds [150]. Additionally, the possibility of generating a cellular skin equivalent is being explored. Hair follicle stem cells have been incorporated into products, such as Integra, to investigate wound healing [151]. A cultured skin substitute using adipose stem cells and keratinocytes has been developed that produces epidermal, dermal, and hypodermal stratification [152]. Moreover, human adipose stem cells that would normally be discarded have recently been isolated from debrided burn eschar tissue [153] and used to generate a tri-layered, vascularized construct [154]. Promising data with nonembryonic stems cells such as these have stimulated interest into future applications and development, and undoubtedly further investigations will produce exciting results.

## Other considerations and future directions

### Monitoring and predicting wound healing

No new skin-based technology can substitute for careful attention by the burn team to the progress (or lack thereof) of wound healing. The WoundFlow computer software program was developed as an enhancement over the traditional paper Lund–Browder diagram to more accurately quantify and track burn injuries over time [104, 155]. WoundFlow is an electronic mapping program that calculates burn size and tracks wound healing [104, 155]. The ability to accurately track burn wound healing over time will support both clinical care and future studies that compare healing rates and outcomes following different treatments. Notably, this study demonstrated that delayed wound healing was associated with a significantly higher risk of mortality [104, 155].

The ability to predict whether a burn wound will spontaneously heal or not would greatly improve patient care. Furthermore, the ability to uniquely tailor treatment to each individual patient would improve patient outcomes and decrease the time to functional recovery, reducing the overall cost of care. Biomarkers may provide a means to allow for tailored treatments and to give insight into wound healing mechanisms [156–161]. Significant efforts in the search for predictive biomarkers for wound failure have determined that serum cytokines, such as interleukin-3 and 12p70, and serum procalcitonin are independently associated with wound failure [161]. Additional candidates have been identified [158–160] but further work is needed to model complex, temporal serum cytokine profiles into an effective predictor for wound healing. In addition to evaluating serum cytokine profiles, candidate biomarkers have been identified in wound effluent [161], which may be a better medium for predicting local wound healing than cytokines in the circulation [162]. Wound exudate has been shown to contain elevated levels of immunosuppressive and proinflammatory cytokines, such as interleukin-1β, interleukin-2, interleukin-6, and tumor necrosis factor alpha [163]. In fact, dipeptidyl peptidase IV and aminopeptidase have been identified in burn wound exudate with a significantly different ratio from that found in plasma [164]. Other work on local wound biomarkers using biopsies has shown that a host of proteins are upregulated during wound healing [165]. More work is needed to establish a biomarker profile that can accurately predict wound healing and to identify potential novel areas for therapeutic intervention.

In addition to examining burn wounds directly, and the wound exudate, another potential method for examining the ability of burn wounds to heal is non-invasive imaging [166]. To this end, a number of non-invasive imaging techniques have been investigated for their use in determining burn depth. Such techniques include terahertz imaging, spatial-frequency-domain imaging, near-infrared spectroscopic imaging, and reflectance-mode confocal microscopy, among others [167–172]. While many of these techniques have not yet been refined sufficiently for clinical application, the most successful research efforts into imaging techniques for burn wounds examine blood flow, such as laser Doppler imaging and indocyanine green angiography [173]. Laser Doppler imaging provides the most evidence for accurately assessing burn severity [174], but it has been shown that laser Doppler imaging is only superior to visual assessment 48 h after thermal injury [175]. Additional studies are needed to fully explore the potential for incorporation of non-invasive imaging modalities into the routine treatment of burn wounds.

### Obese patients

As the obese population continues to grow [176], new treatment approaches will be required. Obese burn patients present with a variety of unique characteristics that include: increased rates of diabetes, hypertension, cardiac disease, and pulmonary disease; altered pharmacokinetics and pharmacodynamics; and altered immune responses [177]. Even the commonly used Lund–Browder chart for estimation of TBSA is problematic for obese patients because it fails to account for altered body-mass distribution in these patients [178]. Hence, analysis of group differences and controlled clinical studies in unique patient populations are needed [179].

### Older patients

Census predictions suggest that the older population will double in the next 20 years. Since older people are at increased risk for burn injury, an increasing number of burn injuries among the older population should be expected. A recent review delineated the unique burn pathophysiology, comorbidities, and treatment strategies for the older population [180]. Detailing all of the unique considerations for the older burn population is outside the scope of this review, but several key points are noteworthy. Most burns among older people occur at home, especially in the kitchen and bathroom, due to diminished alertness, slower reaction time, and reduced mobility [181]. Reductions in metabolic rate and skin thickness with age result in more severe burns, and more extensive full-thickness burns are associated with increased mortality [182]. Comorbidities such as diabetes and cardiovascular disease complicate treatment, and may exacerbate the postburn hypermetabolic response [183]. Several formulas for predicting the survival of older patients, such as the Baux score [184], have received wide acceptance and can help guide clinicians in patient treatment. Unique treatment considerations for older patients should include attentive resuscitation to reduce the risk of volume overload, judicious ventilator support, careful analgesic administration, prudently timed excision and grafting, and extended rehabilitation for functional recovery [180]. The older population presents a unique challenge to the burn clinician, and the treatment of patients must be carefully considered on a case-by-case basis.

### Future directions

Adult burn patients with increased markers of inflammatory stress exhibit reduced serum levels of vitamin A despite normal markers of oxidative stress [185–187]. Additionally, limited preclinical studies show that polyprenoic acid and retinol can facilitate wound healing [188], and that retinoids are efficacious on a variety of other skin conditions [189]. Moreover, early clinical studies have shown that retinoid treatment effectively increases scar elasticity [190, 191]. Taken together, these data highlight the need for studies evaluating retinoids on burn wound healing outcomes.

Pirfenidone was originally developed as an antihelminthic and antipyretic agent, but recent work has demonstrated that it also has anti-inflammatory, antioxidative, and antiproliferative effects [192]. In particular, the antifibrotic properties of pirfenidone attenuate fibroblast proliferation and collagen deposition in vitro and in preclinical models [192]. Pirfenidone is approved for the treatment of idiopathic pulmonary fibrosis in Europe, Japan, and the USA. The antifibrotic actions of pirfenidone and other data suggest that pirfenidone could modulate the tissue response to injury at multiple stages of wound repair to improve scarring and function as an adjuvant for abnormal wound healing processes. Preclinical investigations are currently underway in rabbits [193, 194] and rats [195], but controlled clinical studies are needed to evaluate the safety and efficacy of pirfenidone on abnormal wound healing.

The treatment of burn wounds with hyperbaric oxygen was first investigated in the mid-1960s and garnered some attention in the decades following, but controversy remains over potential risks and costs [196, 197]. Recent work in rat models has shown that hyperbaric oxygen reduces healing time and improves scar appearance of burn injuries [198]. Advancements in hyperbaric chambers have reduced the overall cost associated with treatment, and controlled clinical trials in humans are beginning to produce data supporting the conclusion that hyperbaric oxygen is safe and effective for improving burn wound healing [199–201]. However, more data are needed before broad conclusions can be made about the overall utility of hyperbaric oxygen for treating burns.

Future research on burn patient care will focus on a variety of areas [202]. Considering a current survival rate of over 97 % for burn patients [3], major advancements from the past several decades have improved patient care such that significant future improvements in patient survival rate will be more difficult. However, improvements are still needed in individualized care, namely prediction of patient outcomes and the ability to tailor treatment to optimize functional recovery. Improvements are also needed to accelerate wound closure and healing and to improve psychological care to promote successful reintegration. Research in inflammation, infection, stem cells, grafting, biomarkers, inflammation control, and rehabilitation will continue to improve individualized care and create new treatment options.

## Conclusion

The various clinical challenges in treating acute thermal injuries include balancing the many factors that affect wound healing to reduce the length of stay (and associated cost of treatment), the risk of infection, the time to wound closure, and the overall time to functional recovery. The treatment of burn wounds has evolved over several decades through clinical and preclinical research. Significant advancements have been made in patient care, including tracking wound healing, developing novel graft and coverage options, controlling inflammation, optimizing dietary needs, and testing unique pharmacological interventions. As a result of these efforts, patient survival has improved along with a concomitant decrease in the length of stay, which in turn results in a decreased cost to the patient and the medical providers. A summary of selected clinical recommendations is provided (Table 3) to aid the intensivist, but it is important to remember that burn patients present unique challenges based on multiple variables (for example, age, TBSA, comorbidities) and treatment decisions must be tailored to each patient’s needs. Current and future research will continue to identify novel targets and treatment paradigms to further improve burn wound care.

**Table 3:** Recommendations for the intensivist

Accurate measurement of burn size using a Lund–Browder chart
Carefully titrated fluid resuscitation, to balance risks of edema formation with those of ongoing hypoperfusion
Early initiation of effective topical antimicrobial therapy (mafenide acetate or silver-based creams/dressings)
Daily inspection of the wounds by a qualified surgeon or wound care expert
Early excision and grafting of all full thickness and deep partial thickness burns
Aggressive treatment of infected wounds (resuscitate, broad-spectrum topical and systemic antimicrobials, excision, or re-excision)
Rehabilitation in the ICU to minimize the functional consequences of prolonged immobilization and contracture formation

## Abbreviation

TBSA: Total body surface area
